# Crystal structure, Hirshfeld surface analysis, DFT optimized mol­ecular structure and the mol­ecular docking studies of 1-[2-(cyano­sulfan­yl)acet­yl]-3-methyl-2,6-bis­(4-methyl­phen­yl)piperidin-4-one

**DOI:** 10.1107/S2056989024008508

**Published:** 2024-09-12

**Authors:** A. R. Karthiga, S. Divyabharathi, R. Reshwen Shalo, K. Rajeswari, T. Vidhyasagar, S. Selvanayagam

**Affiliations:** a Department of Chemistry Annamalai University, Annamalainagar Chidambaram 608 002 India; b Department of Chemistry Annamalai University, Annamalainagar Chidambaram 608 002 PG & Research Department of Chemistry Government Arts College Chidambaram 608 102 India; c PG & Research Department of Physics, Government Arts College, Melur 625 106, India; University of Buenos Aires, Argentina

**Keywords:** crystal structure, piperidine derivatives, superposition, C—H⋯O intra­molecular inter­actions, C—H⋯O inter­molecular hydrogen bonds, Hirshfeld surface analysis

## Abstract

The two mol­ecules in the asymmetric unit of the title compound, C_23_H_24_N_2_O_2_S, have a structural overlap with an r.m.s. deviation of 0.82 Å. The piperidine rings adopt a distorted boat conformation. Intra- and inter­molecular C—H⋯O hydrogen bonds are responsible for the cohesion of the crystal packing. The inter­molecular inter­actions were qu­anti­fied and analysed using Hirshfeld surface analysis. The mol­ecular structure optimized by density functional theory (DFT) at the B3LYP/6–311++G(d,p)level is compared with the experimentally determined mol­ecular structure in the solid state.

## Abstract 2

In the title compound, C_23_H_24_N_2_O_2_S, the two mol­ecules in the asymmetric unit have a structural overlap with an r.m.s. deviation of 0.82 Å.

## 1. Chemical context

Organic thio­cyanates (*R*SCN) are important synthetic inter­mediates for accessing various valuable sulfur-containing compounds. They belong to the chemical class of organic chalcogen-cyanates (*R*—*X*—C*R*N), in which the heteroatom *X* (*i.e.* O, S, Se, Te) is attached by a single bond to the organic substituent (alkyl, ar­yl⋯) and by another one to the CN group. As a result of the specific reactivity of the *X*CN function (particularly when acting as a leaving group), these compounds are often considered organic pseudohalides (Castanheiro *et al.*, 2016 ▸; Chen *et al.*, 2022 ▸). Organic thio­cyanates exhibit a wide spectrum of biological activities such as anti­proliferative (Kumar *et al.*, 2014 ▸), anti­cancer (Krishnegowda *et al.*, 2011 ▸), cytotoxic (Noolvi *et al.*, 2011 ▸), the causative agent of Chagas’ disease (Liñares *et al.*, 2007 ▸) and treatment of leishmanial infections (Cottrell *et al.*, 2004 ▸).
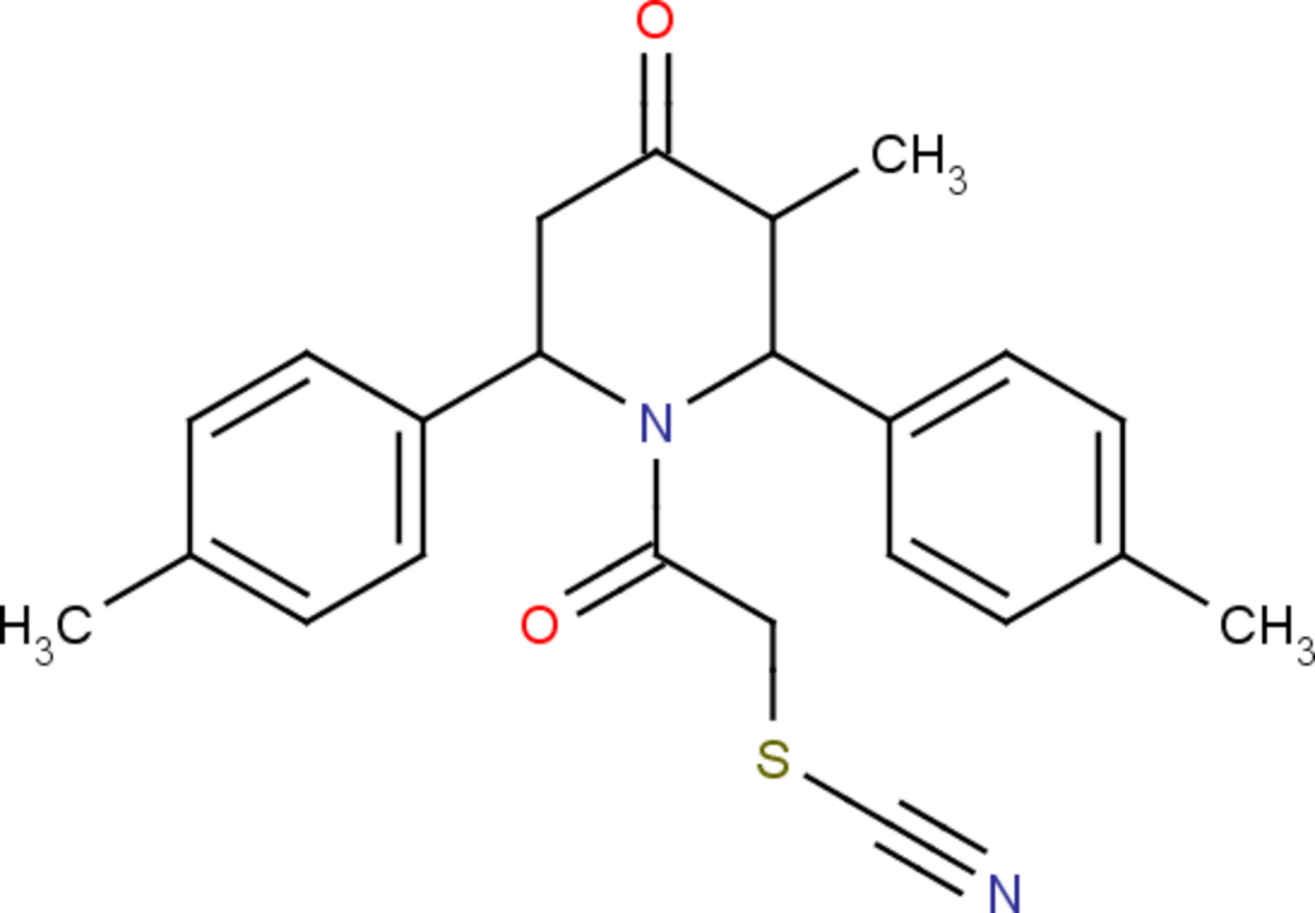


In view of the importance of such compounds, we have undertaken a single-crystal X-ray diffraction study of the title compound and the results are presented here. In addition, DFT, Hirshfeld surface and mol­ecular docking studies were carried out to determine the electronic properties, inter­molecular contacts and protein–ligand inter­actions of the compound.

## 2. Structural commentary

**Figure 1:**
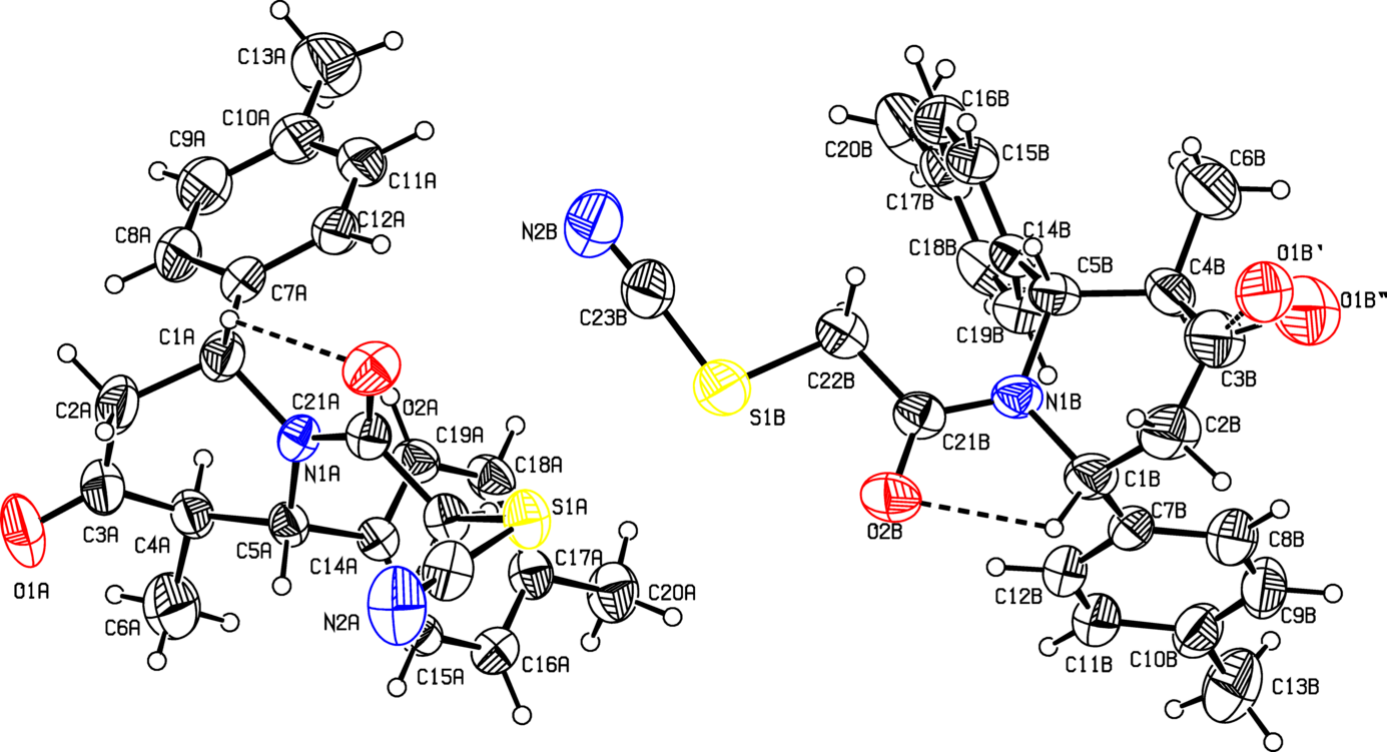
A view of the mol­ecular structure of compound (I), showing the atom labelling. Displacement ellipsoids are drawn at the 30% probability level. Intra­molecular hydrogen bonds are shown as dashed lines.

**Figure 2:**
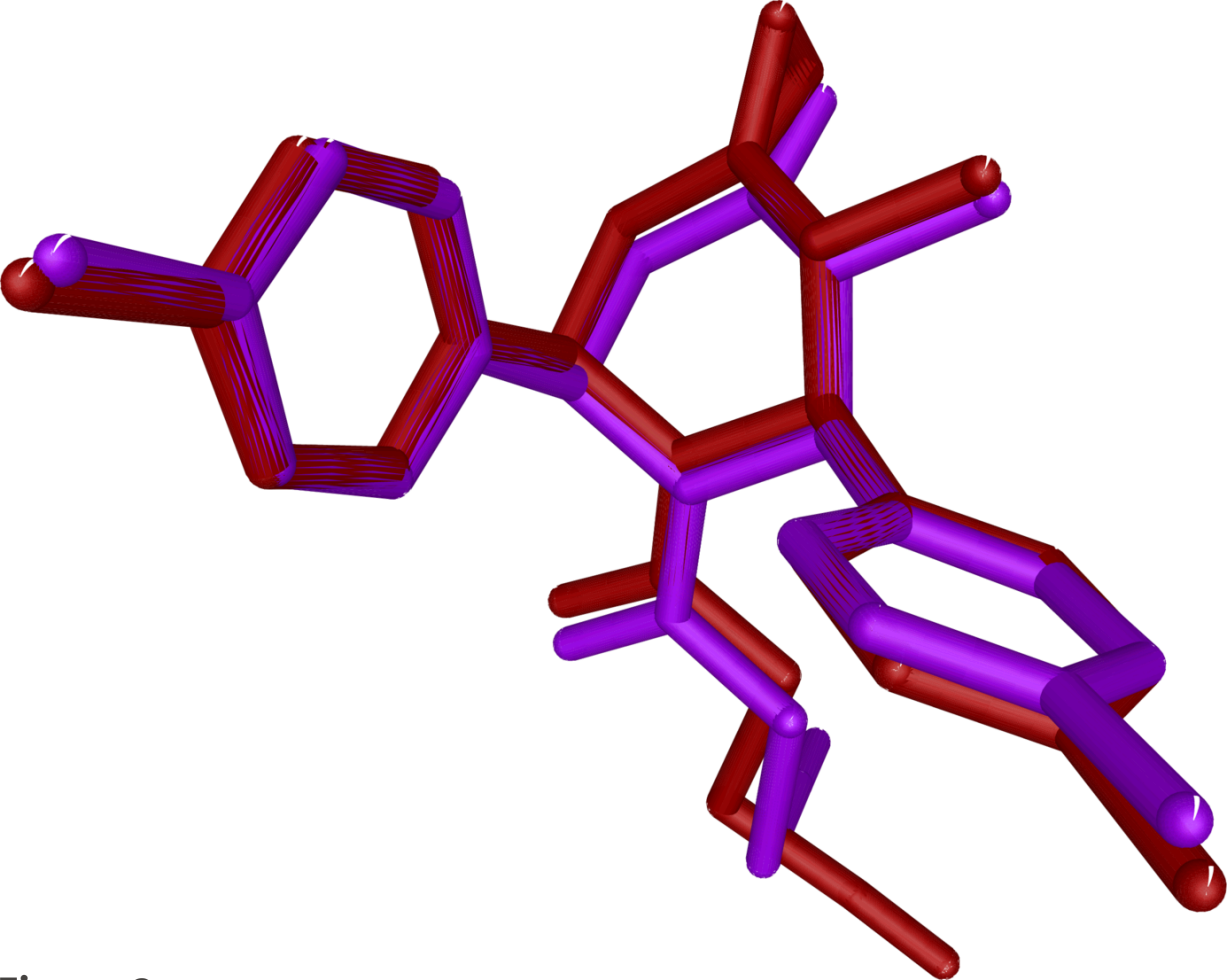
Superposition of mol­ecule *A* (purple) and mol­ecule *B* (brown) in compound (I) except for O1.

The mol­ecular structure of the title compound, (I), is illus­trated in Fig. 1 ▸. There are two mol­ecules in the asymmetric unit, *A* and *B*. Fig. 2 ▸ shows a superposition of the two mol­ecules except for O1 (due to disorder of this atom in molecule *B*) using *Qmol* (Gans & Shalloway, 2001 ▸); the r.m.s. deviation is 0.82 Å. The two methyl­phenyl rings in mol­ecule *A* are oriented at a dihedral angle of 74.6 (1)°. The methyl atoms C13 and C20 in mol­ecule *A* deviate by −0.043 (1) and 0.018 (1) Å, respectively, from the ring to which they are attached. The two methyl­phenyl rings in mol­ecule *B* subtend a dihedral angle of 68.0 (1)°. The methyl atoms C13 and C20 in mol­ecule *B* deviate by −0.013 (1) and 0.035 (1) Å, respectively, from the ring to which they are attached. The piperidine rings (N1/C1–C5) in both mol­ecules *A* and *B* have a distorted boat conformation, with puckering parameters (Cremer & Pople, 1975 ▸) *q*_2_ = *Q*_T_ = 0.669 (1) Å and θ = 90.6 (1)° (mol­ecule *A*) and *q*_2_ = *Q*_T_ = 0.676 (1) Å and θ = 92.5 (1)° (mol­ecule *B*). An intra­molecular C—H⋯O contact leads to the stabilization of the mol­ecular conformation in both *A* and *B* (Fig. 1 ▸ and Table 1 ▸).

## 3. Supra­molecular features

**Figure 3:**
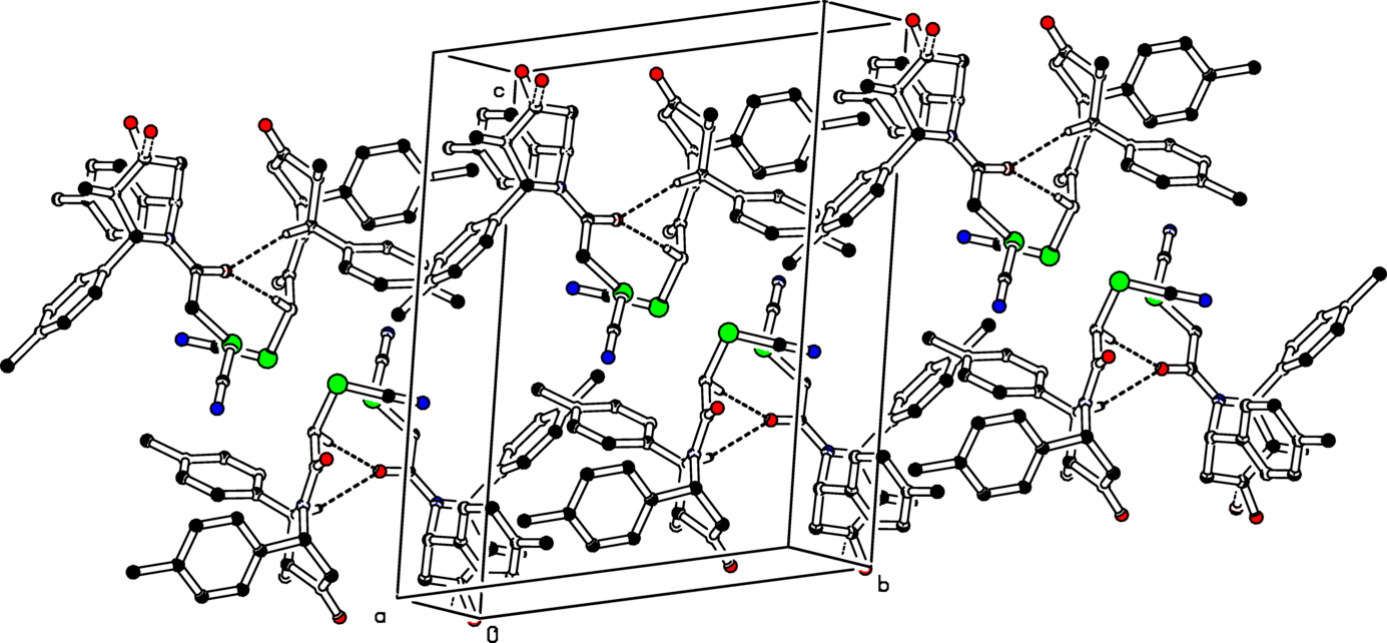
The crystal packing of the title compound (I) viewed down the *a* axis. The C—H⋯O inter­molecular inter­actions are shown as dashed lines. For clarity H atoms not involved in these hydrogen bonds have been omitted.

**Figure 4:**
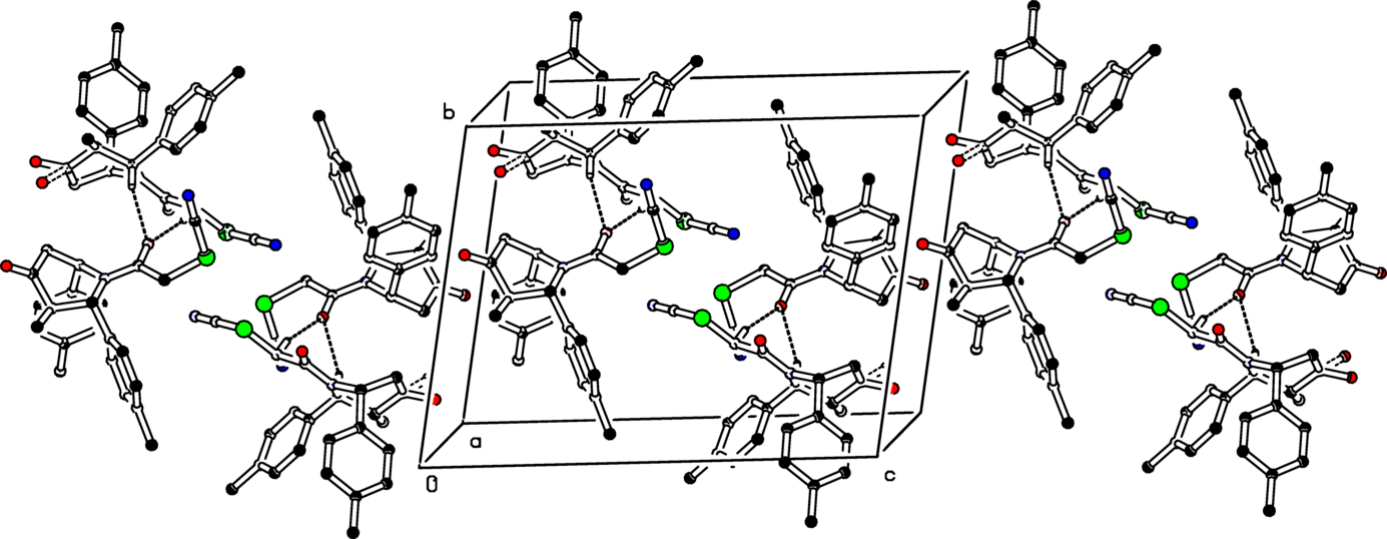
The crystal packing of the title compound (I) showing C—H⋯O inter­molecular inter­actions as dashed lines. For clarity H atoms not involved in these hydrogen bonds have been omitted.

In the crystal of compound (I), mol­ecules *A* associate with *B* mol­ecules *via* C—H⋯O inter­actions (C5*A*—H5*A*⋯O2*B*^i^ and C22*A*—H22*B*⋯O2*B*^i^; Table 1 ▸) propagating along [110] in an anti-parallel manner, see Fig. 3 ▸. Similarly, *B* mol­ecules associate with *A* mol­ecules *via* C—H⋯O inter­actions (C5*B*—H5*B*⋯O2*A*^ii^ and C22*B*—H22*D*⋯O2*A*^ii^, Table 1 ▸) propagating along [110] in an anti-parallel manner; see Fig. 4 ▸. Atoms O2*A* and O2*B* act as bifurcated acceptors for these inter­molecular inter­actions.

## 4. Hirshfeld surface analysis

**Figure 5:**
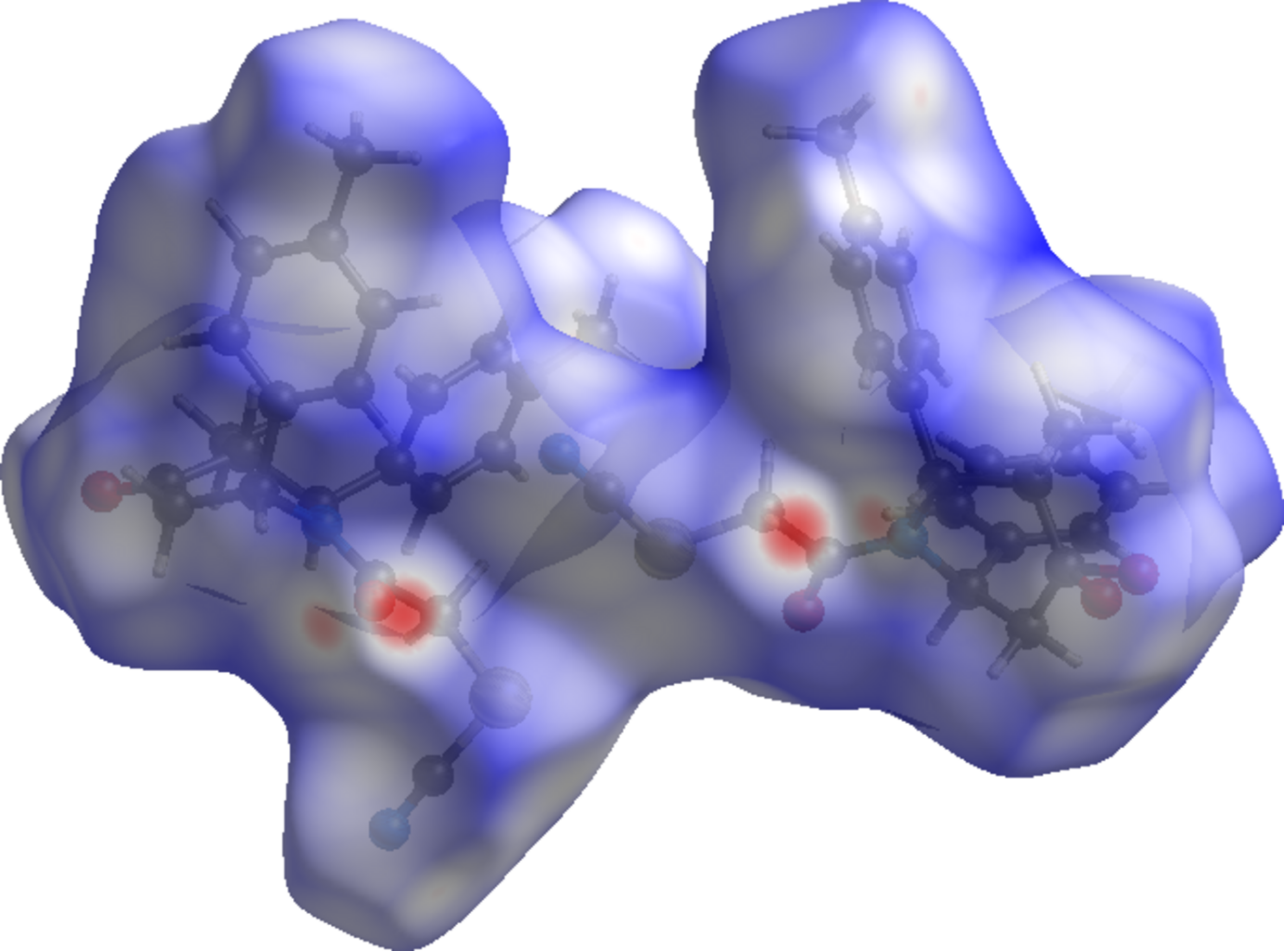
A view of the Hirshfeld surface mapped over *d*_norm_.

To further characterize the inter­molecular inter­actions in the title compound, we carried out a Hirshfeld surface (HS) analysis (Spackman & Jayatilaka, 2009 ▸) using *Crystal Explorer 21* (Spackman *et al.*, 2021 ▸) and generated the associated two-dimensional fingerprint plots (McKinnon *et al.*, 2007 ▸). The HS mapped over *d*_norm_ in the range −0.3611 to +1.5697 a.u. is illustrated in Fig. 5 ▸, using colours to indicate contacts that are shorter (red areas), equal to (white areas), or longer than (blue areas) the sum of the van der Waals radii (Ashfaq *et al.*, 2021 ▸).

**Figure 6:**
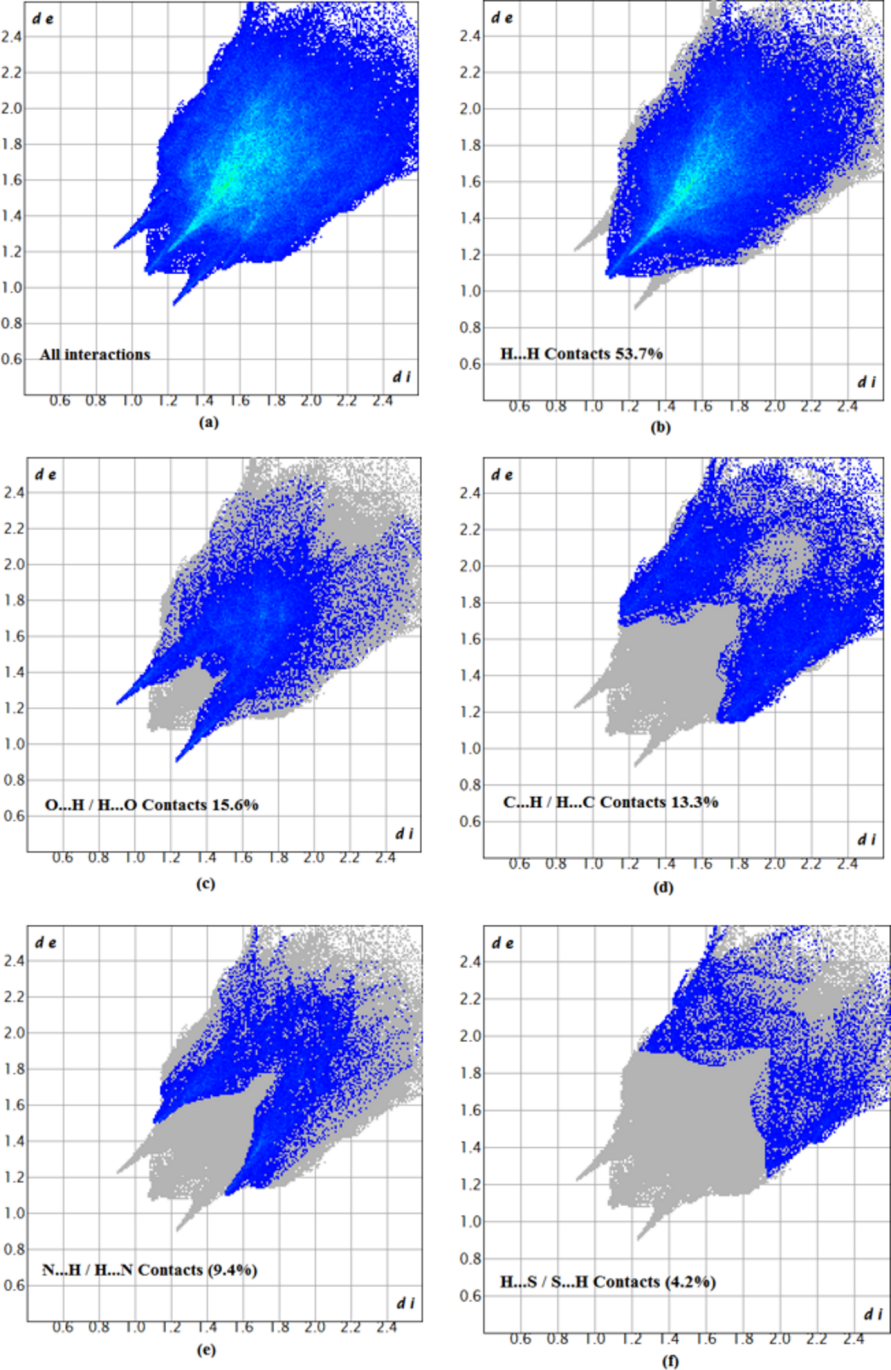
Two-dimensional fingerprint plots for the compound (I), showing (*a*) all inter­actions, and delineated into (*b*) H⋯H, (*c*) O⋯H/H⋯O, (*d*) C⋯H/H⋯C, (*e*) N⋯H/H⋯N and (*f*) S⋯H/H⋯S inter­actions. The *d*_i_ and *d*_e_ values are the closest inter­nal and external distances (in Å) from given points on the Hirshfeld surface.

The two-dimensional fingerprint plots provide qu­anti­tative information about the non-covalent inter­actions and the crystal packing in terms of the percentage contribution of the inter­atomic contacts (Spackman & McKinnon, 2002 ▸; Ashfaq *et al.*, 2021 ▸). The overall two-dimensional fingerprint plot is shown in Fig. 6 ▸*a*. The HS analysis reveals that H⋯H (53.7%) and H⋯O/O⋯H (15.6%) contacts are the main contributors to the crystal packing, followed by H⋯C/C⋯H (13.3%), N⋯H/H⋯N (9.4%) and H⋯S/S⋯H (4.2%) contacts; see Fig. 6 ▸*b*–*f*. The HS analysis confirms the importance of H-atom contacts in establishing the packing. The large number of H⋯H, H⋯O/O⋯H and H⋯C/C⋯H inter­actions suggest that van der Waals and C—H⋯O hydrogen-bonding inter­actions in the structure play the major roles in the crystal packing (Hathwar *et al.*, 2015 ▸). The fragment patches on the HS provide an easy way to investigate the nearest neighbour coordination environment of a mol­ecule (coordination number), which is 23.

## 5. DFT Studies

**Figure 7:**
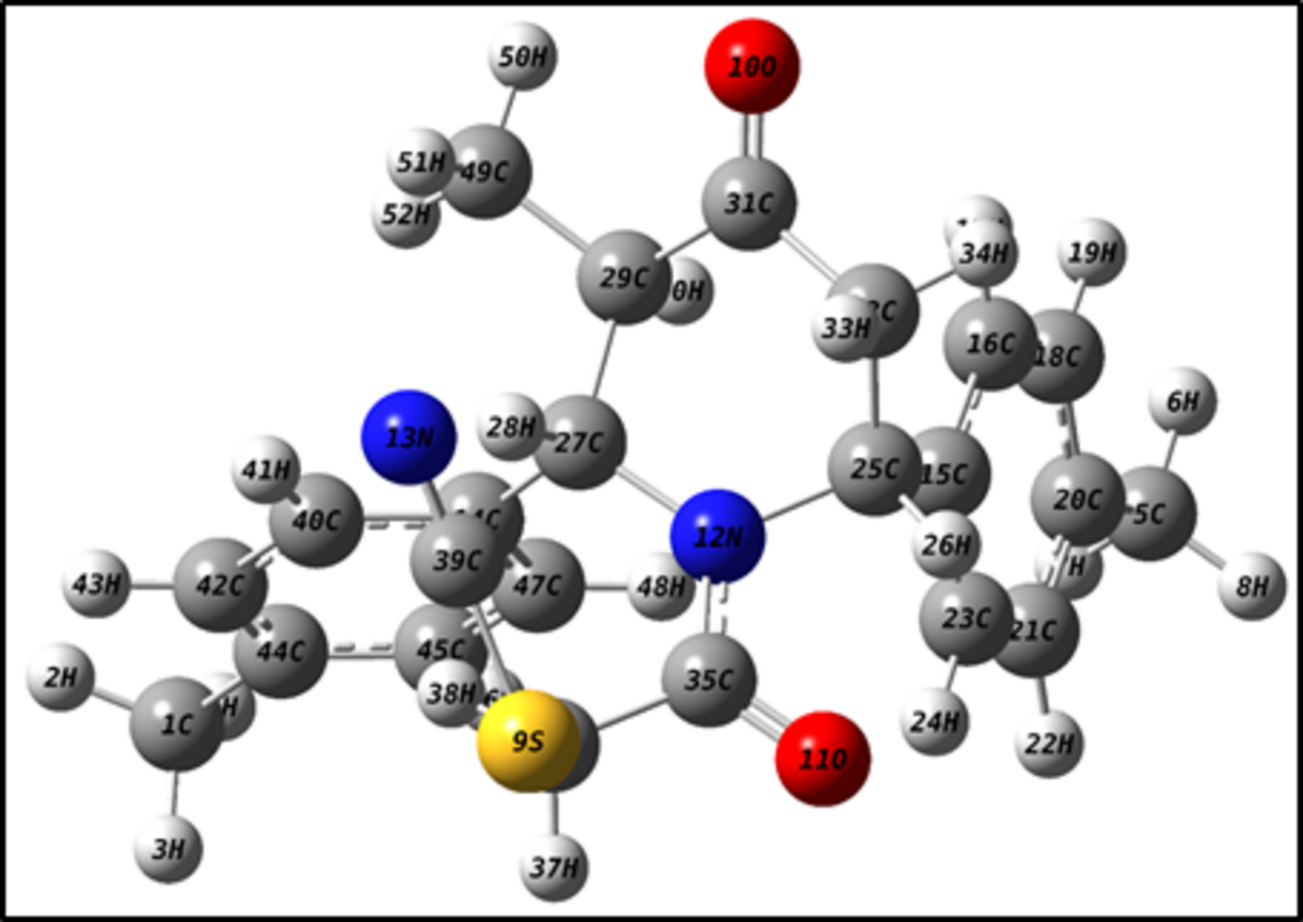
DFT optimized structure of (I).

**Figure 8:**
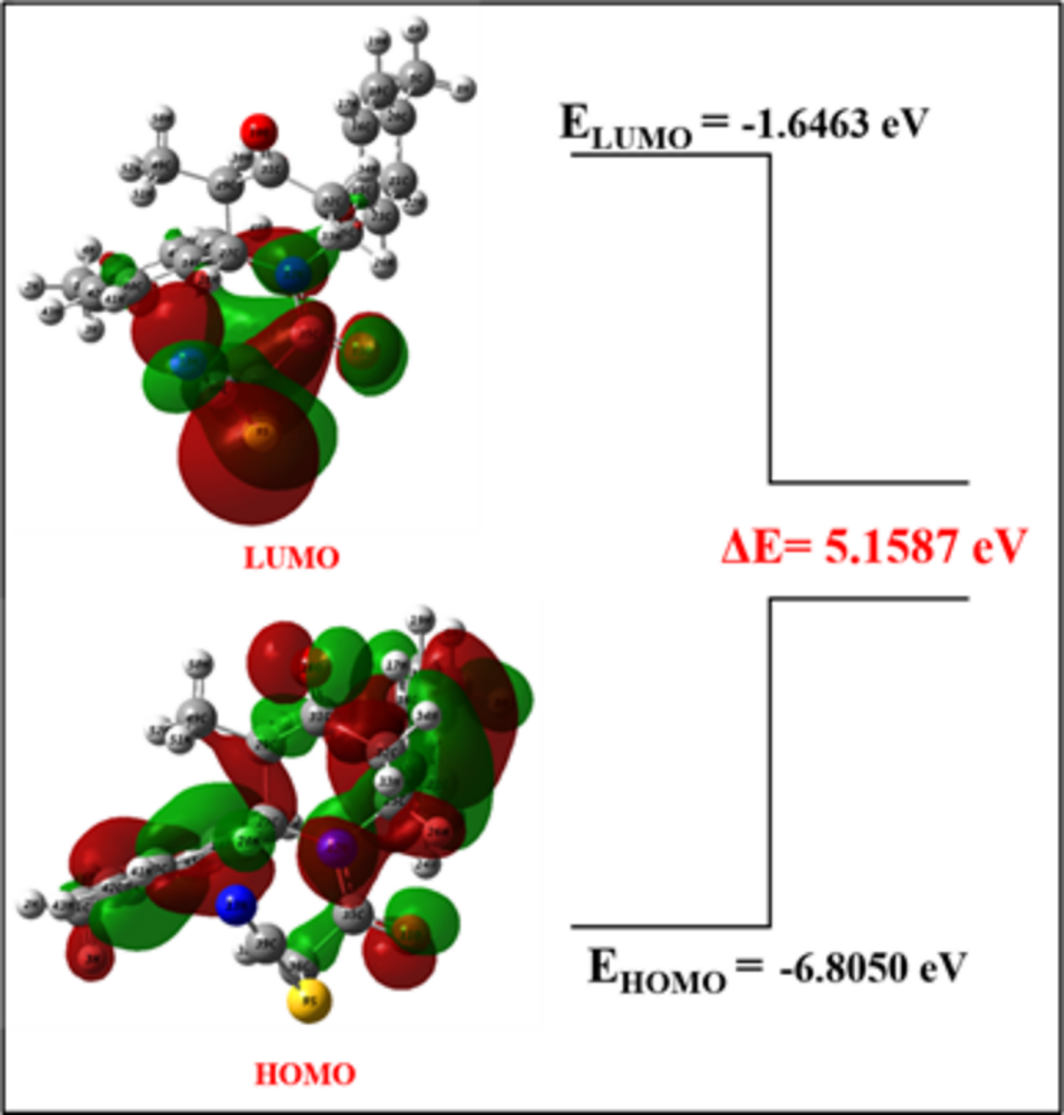
Frontier mol­ecular orbital structure of (I).

**Figure 9:**
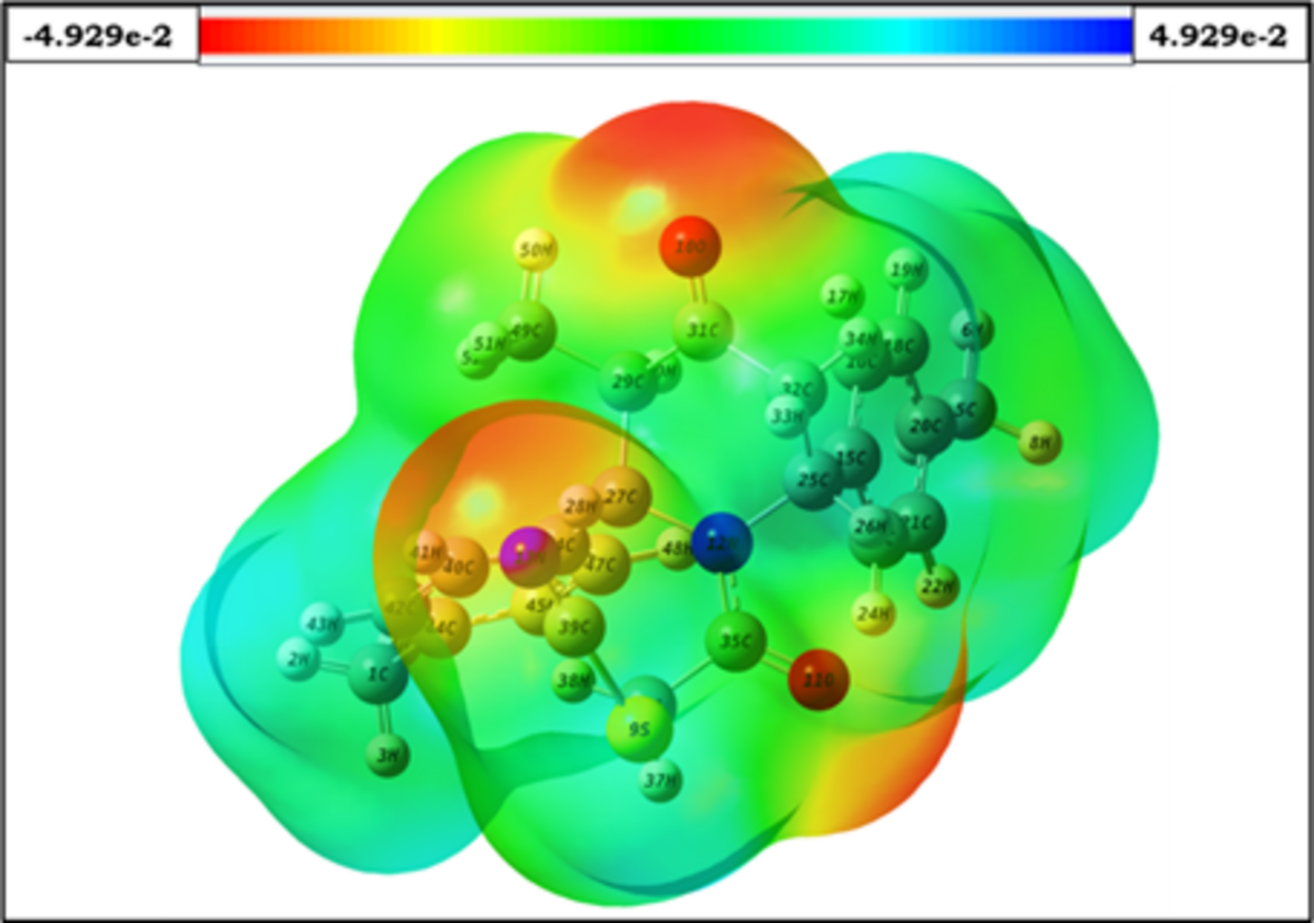
Mol­ecular electrostatic potential surface of (I).

The optimized structure of (I) in the gas phase was computed using DFT at the B3LYP/6-311++ G(d,p) level of theory with *Gaussian 09W* (Frisch, 2009 ▸), and *GaussView 5.0* was used to generate the optimized structure (Fig. 7 ▸), the HOMO and LUMO (Fig. 8 ▸) and the MEP surface (Fig. 9 ▸). The optimized structure reveals the conformation of the piperidine ring of (I) as a distorted boat in the gas phase, which is concordant with the findings obtained from the SC-XRD (solid state) study. Comparison of theoretical bond parameters with those obtained from the diffraction study show the consistency between them (Table 2 ▸).

The frontier mol­ecular orbitals HOMO and LUMO of compound (I) were computed using DFT [B3LYP/6-311++ G(d,p) method]. The calculated energies of the HOMO and LUMO are −6.8050 and −1.6463 eV, respectively. The energy gap Δ*E* is 5.1587 eV. The value of Δ*E* can also be utilized to understand the biological activity (Behzadi *et al.*, 2015 ▸; Gülseven Sidir *et al.*, 2011 ▸), *i.e*., lower toxicity, longer half-life and sustained activity can be correlated and understood from the value of Δ*E*. Therefore, compound (I) with Δ*E =* 5.1587 eV is expected to have a pronounced biological influence with minimum side effects.

The MEP surface of the optimized structure of (I) is depicted in Fig. 9 ▸. Nucleophilic and electrophilic reactive sites of the mol­ecule are represented by red- and blue-coloured regions on the MEP surface. In the MEP surface of (I), the red colour covers both carbonyl oxygen atoms and the nitro­gen atom of the thio­cyanate group, revealing their sensitivity towards nucleophilic attack. The pale-blue colour spread over the phenyl rings indicates weak electrophilic sites. The existence of these areas on the MEP surface predicts the favourable inter­action sites of the mol­ecule (towards chemical reactions and binding sites for targeted biological entities (Rathi *et al.*, 2020 ▸).

## 6. Mol­ecular docking studies

Among the numerous life-threatening types of cancers, multiple reports have emphasized that more than one in ten new cancer cases diagnosed in women worldwide are identified as breast cancer. The development and progression of breast cancer can be controlled by targeting ERα receptors, as these receptors only get activated when they get bound with estradiol, an estrogen hormone. Drugs like tamoxifen and doxorubicin bind to ER and block the action of estrogen, thus inhibiting the action of these receptors and thereby cancerous growth (Li *et al.*, 2011 ▸).

**Figure 10:**
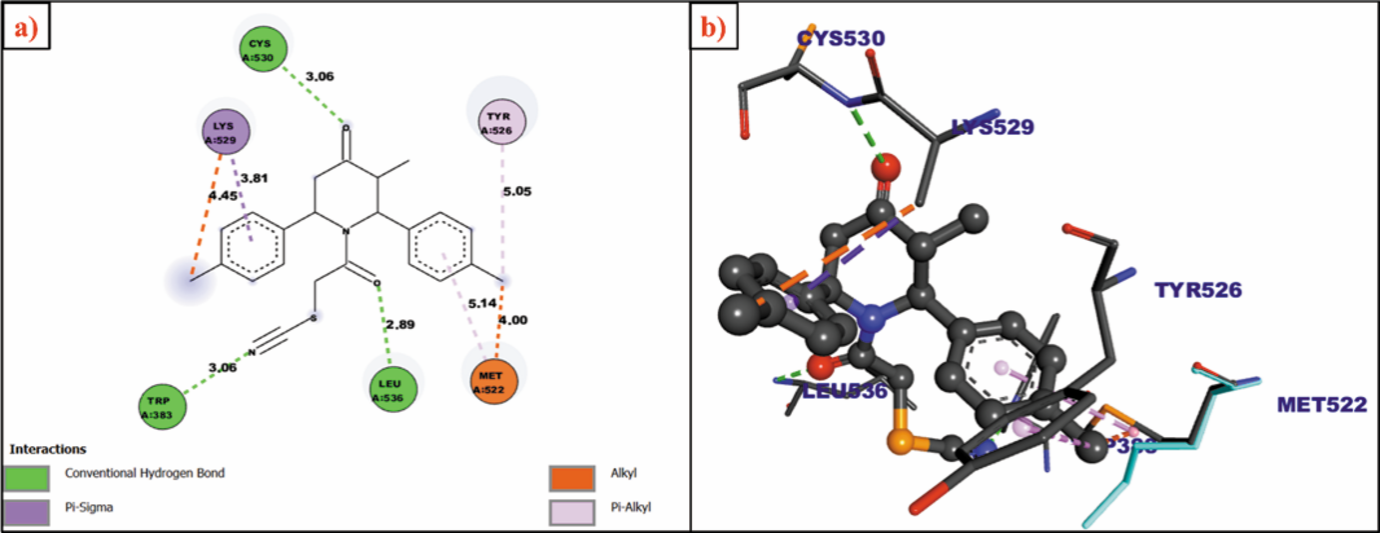
Two- and three-dimensional inter­actions of the title compound (ligand) with amino acid residues with distances in Å.

The human estrogen receptor is a type of nuclear receptor with a PDB ID: 3ERT that was chosen for the present docking study to explore the anti­cancer potency of the title compound (I). Mol­ecular docking by *AutoDock* tools (Huey *et al.*, 2012 ▸; Ferreira *et al.*, 2015 ▸) was used to predict the binding efficiency of ligand mol­ecule (I) with the target protein (3ERT) (Fig. 10 ▸). To compare the efficacy of the mol­ecule under study, its binding affinity was also compared with those of two standard drugs, *viz*., tamoxifen and doxorubicin whose binding energies were calculated by adopting similar procedure as that for the title compound. Surprisingly, the binding affinity of the title compound (I) towards 3ERT (−9.11 kcal mol^−1^) is comparable with that of tamoxifen (−8.02 kcal mol^−1^) and doxorubicin (−10.02 kcal mol^−1^). Among the several inter­actions of the ligand (I) with the target protein, the conventional hydrogen-bonding inter­actions seen between three moieties, *i.e.*, two carbonyl oxygen atoms and the nitro­gen atom of the thio­cyanato group with three different amino acid groups (CYS A:530, LEU A:536, TRP A:383) attracts inter­est because the MEP surface diagram (Fig. 9 ▸) also highlights these three areas of the mol­ecule as electron-rich centres that are vulnerable sites for nucleophilic attacks, which is emphasized by the docking at these sites.

## 7. Synthesis and crystallization

Compound (I) was synthesized by adopting the procedure previously reported by us (Pillai *et al.*, 2016 ▸). The solid product was collected, washed and recrystallized from methanol to obtain the pure product.

## 8. Refinement

Crystal data, data collection and structure refinement details are summarized in Table 3 ▸. H atoms were placed in idealized positions and allowed to ride on their parent atoms: C—H = 0.93–0.98 Å, with *U*_iso_(H) = 1.5*U*_eq_(C-meth­yl) and 1.2*U*_eq_(C) for other H atoms. Atom O1 in mol­ecule *B* is disordered over two positions, with the occupancy of the major component being 0.58 (12).

## Supplementary Material

Crystal structure: contains datablock(s) I, global. DOI: 10.1107/S2056989024008508/vu2005sup1.cif

Structure factors: contains datablock(s) I. DOI: 10.1107/S2056989024008508/vu2005Isup2.hkl

Supporting information file. DOI: 10.1107/S2056989024008508/vu2005Isup3.cml

CCDC reference: 2380151

Additional supporting information:  crystallographic information; 3D view; checkCIF report

## Figures and Tables

**Table 1:** Hydrogen-bond geometry (Å, °)

*D*—H⋯*A*	*D*—H	H⋯*A*	*D*⋯*A*	*D*—H⋯*A*
C1*A*—H1*A*⋯O2*A*	0.98	2.22	2.706 (5)	109
C1*B*—H1*B*⋯O2*B*	0.98	2.20	2.697 (6)	110
C5*A*—H5*A*⋯O2*B*^i^	0.98	2.40	3.345 (5)	161
C5*B*—H5*B*⋯O2*A*^ii^	0.98	2.51	3.458 (5)	163
C22*A*—H22*B*⋯O2*B*^i^	0.97	2.24	3.162 (5)	159
C22*B*—H22*D*⋯O2*A*^ii^	0.97	2.34	3.293 (5)	166

Symmetry codes: (i) 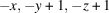; (ii) 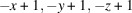.

**Table 2:** Selected bond distances, bond angles and torsion angles (Å, °) and theoretical (DFT) calculations of mol­ecules *A* and *B*

Parameter	Mol­ecule *A*	Mol­ecule *B*	DFT
S1—C22	1.802 (4)	1.785 (4)	1.857
S1—C23	1.670 (6)	1.660 (7)	1.698
N2—C23	1.133 (6)	1.149 (7)	1.16
O2—C21	1.221 (5)	1.226 (5)	1.21
N1—C21	1.357 (5)	1.350 (5)	1.225
O1—C3	1.206 (6)	1.25 (2)	1.371
O2—C21—N1	122.8 (4)	122.3 (4)	122.918
O1—C3—C4	122.1 (6)	124.8 (12)	122.429
N2—C23—S1	177.9 (5)	177.2 (6)	178.413
N1—C21—C22	116.9 (4)	119.1 (4)	119.705
C21—C22—S1	114.3 (3)	106.7 (3)	111.057
C3—C4—C5—C14	−173.2 (4)	−174.4 (4)	−170.027
C7—C1—C2—C3	78.6 (5)	74.5 (6)	74.64
C3—C4—C5—N1	−46.7 (5)	−47.4 (5)	−44.249
N1—C1—C2—C3	−47.9 (5)	−53.2 (7)	−54.725
O1—C3—C4—C5	−132.8 (6)	−114 (4)	−145.373
C1—C2—C3—O1	−178.3 (5)	168 (3)	−164.24
O1—C3—C4—C6	−7.6 (7)	12 (4)	−17.713
C6—C4—C5—N1	−171.0 (4)	−173.6 (5)	−171.39

**Table 3:** Experimental details

Crystal data	
Chemical formula	C_23_H_24_N_2_O_2_S
*M*_r_	392.50
Crystal system, space group	Triclinic, *P* 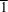
Temperature (K)	300
*a*, *b*, *c* (Å)	9.3404 (6), 13.1888 (9), 17.4549 (11)
α, β, γ (°)	79.515 (2), 87.981 (2), 88.516 (2)
*V* (Å^3^)	2112.6 (2)
*Z*	4
Radiation type	Mo *K*α
μ (mm^−1^)	0.17
Crystal size (mm)	0.20 × 0.15 × 0.11

Data collection	
Diffractometer	Bruker APEXII CCD
No. of measured, independent and observed [*I* > 2σ(*I*)] reflections	43593, 7726, 3914
*R*_int_	0.068
(sin θ/λ)_max_ (Å^−1^)	0.602

Refinement	
*R*[*F*^2^ > 2σ(*F*^2^)], *wR*(*F*^2^), *S*	0.077, 0.242, 1.05
No. of reflections	7726
No. of parameters	515
H-atom treatment	H-atom parameters constrained
Δρ_max_, Δρ_min_ (e Å^−3^)	0.64, −0.37

Computer programs: *APEX2* and *SAINT* (Bruker, 2012 ▸), *SHELXT2018/2* (Sheldrick, 2015*a* ▸), *ORTEP-3 for Windows* (Farrugia, 2012 ▸), *SHELXL2019/2* (Sheldrick, 2015*b* ▸) and *PLATON* (Spek, 2020 ▸).

## supplementary crystallographic information

### 1-[2-(Cyanosulfanyl)acetyl]-3-methyl-2,6-bis(4-methylphenyl)piperidin-4-one Crystal data

**Table:**

C_23_H_24_N_2_O_2_S	*Z* = 4
*M**_r_* = 392.50	*F*(000) = 832
Triclinic, *P*1	*D*_x_ = 1.234 Mg m^−^^3^
*a* = 9.3404 (6) Å	Mo *K*α radiation, λ = 0.71073 Å
*b* = 13.1888 (9) Å	Cell parameters from 8307 reflections
*c* = 17.4549 (11) Å	θ = 2.4–19.2°
α = 79.515 (2)°	µ = 0.17 mm^−^^1^
β = 87.981 (2)°	*T* = 300 K
γ = 88.516 (2)°	Block, yellow
*V* = 2112.6 (2) Å^3^	0.20 × 0.15 × 0.11 mm

### 1-[2-(Cyanosulfanyl)acetyl]-3-methyl-2,6-bis(4-methylphenyl)piperidin-4-one Data collection

**Table:**

Bruker APEXII CCD diffractometer	*R*_int_ = 0.068
φ and ω scans	θ_max_ = 25.4°, θ_min_ = 1.8°
43593 measured reflections	*h* = −11→11
7726 independent reflections	*k* = −15→15
3914 reflections with *I* > 2σ(*I*)	*l* = −21→21

### 1-[2-(Cyanosulfanyl)acetyl]-3-methyl-2,6-bis(4-methylphenyl)piperidin-4-one Refinement

**Table:**

Refinement on *F*^2^	0 restraints
Least-squares matrix: full	Hydrogen site location: inferred from neighbouring sites
*R*[*F*^2^ > 2σ(*F*^2^)] = 0.077	H-atom parameters constrained
*wR*(*F*^2^) = 0.242	*w* = 1/[σ^2^(*F*_o_^2^) + (0.1031*P*)^2^ + 1.1564*P*] where *P* = (*F*_o_^2^ + 2*F*_c_^2^)/3
*S* = 1.05	(Δ/σ)_max_ < 0.001
7726 reflections	Δρ_max_ = 0.64 e Å^−^^3^
515 parameters	Δρ_min_ = −0.37 e Å^−^^3^

### 1-[2-(Cyanosulfanyl)acetyl]-3-methyl-2,6-bis(4-methylphenyl)piperidin-4-one Special details

**Table:**

Geometry. All esds (except the esd in the dihedral angle between two l.s. planes) are estimated using the full covariance matrix. The cell esds are taken into account individually in the estimation of esds in distances, angles and torsion angles; correlations between esds in cell parameters are only used when they are defined by crystal symmetry. An approximate (isotropic) treatment of cell esds is used for estimating esds involving l.s. planes.

### 1-[2-(Cyanosulfanyl)acetyl]-3-methyl-2,6-bis(4-methylphenyl)piperidin-4-one Fractional atomic coordinates and isotropic or equivalent isotropic displacement parameters (Å^2^)

**Table:**

	*x*	*y*	*z*	*U*_iso_*/*U*_eq_	Occ. (<1)
S1A	0.12113 (14)	0.61899 (9)	0.46084 (6)	0.0789 (4)	
O1A	−0.0429 (5)	0.6285 (4)	0.0388 (3)	0.164 (2)	
O2A	0.2923 (3)	0.6402 (2)	0.31469 (18)	0.0869 (9)	
N1A	0.1540 (3)	0.5582 (2)	0.24215 (18)	0.0591 (8)	
N2A	0.0045 (6)	0.8171 (4)	0.4120 (3)	0.1205 (17)	
C1A	0.2541 (5)	0.5883 (3)	0.1739 (2)	0.0744 (12)	
H1A	0.318834	0.638910	0.187825	0.089*	
C2A	0.1661 (6)	0.6452 (4)	0.1057 (3)	0.0937 (16)	
H2A1	0.140358	0.713377	0.115756	0.112*	
H2A2	0.226271	0.653899	0.058651	0.112*	
C3A	0.0341 (6)	0.5933 (4)	0.0916 (3)	0.0928 (15)	
C4A	0.0012 (5)	0.4954 (3)	0.1466 (2)	0.0698 (11)	
H4A	0.070851	0.442792	0.135437	0.084*	
C5A	0.0179 (4)	0.5070 (3)	0.2325 (2)	0.0560 (10)	
H5A	−0.060896	0.551752	0.245927	0.067*	
C6A	−0.1425 (7)	0.4601 (5)	0.1327 (3)	0.127 (2)	
H6A1	−0.162402	0.397202	0.168354	0.190*	
H6A2	−0.212891	0.511877	0.140524	0.190*	
H6A3	−0.145887	0.448138	0.080151	0.190*	
C7A	0.3473 (5)	0.4970 (4)	0.1586 (2)	0.0716 (12)	
C8A	0.3551 (6)	0.4589 (5)	0.0907 (3)	0.0966 (16)	
H8A	0.301194	0.490517	0.048855	0.116*	
C9A	0.4411 (6)	0.3745 (5)	0.0828 (3)	0.1048 (17)	
H9A	0.443001	0.350644	0.035817	0.126*	
C10A	0.5240 (5)	0.3248 (4)	0.1421 (3)	0.0887 (14)	
C11A	0.5194 (5)	0.3648 (4)	0.2101 (3)	0.0839 (14)	
H11A	0.575462	0.333986	0.251252	0.101*	
C12A	0.4335 (5)	0.4495 (4)	0.2185 (2)	0.0754 (12)	
H12A	0.433539	0.474861	0.264864	0.090*	
C13A	0.6155 (7)	0.2313 (5)	0.1340 (4)	0.133 (2)	
H13A	0.665008	0.207830	0.181490	0.200*	
H13B	0.555811	0.177407	0.123756	0.200*	
H13C	0.684040	0.248980	0.091713	0.200*	
C14A	0.0006 (4)	0.4026 (3)	0.2839 (2)	0.0518 (9)	
C15A	−0.1263 (4)	0.3784 (3)	0.3260 (2)	0.0588 (10)	
H15A	−0.197188	0.429179	0.326309	0.071*	
C16A	−0.1494 (4)	0.2806 (3)	0.3674 (2)	0.0636 (11)	
H16A	−0.235423	0.266720	0.395345	0.076*	
C17A	−0.0478 (5)	0.2032 (3)	0.3682 (2)	0.0658 (11)	
C18A	0.0804 (5)	0.2273 (3)	0.3277 (3)	0.0706 (12)	
H18A	0.151688	0.176584	0.328521	0.085*	
C19A	0.1045 (4)	0.3253 (3)	0.2861 (2)	0.0647 (11)	
H19A	0.191529	0.339561	0.259268	0.078*	
C20A	−0.0733 (6)	0.0957 (3)	0.4132 (3)	0.0915 (15)	
H20A	0.008925	0.052445	0.406956	0.137*	
H20B	−0.089250	0.098309	0.467459	0.137*	
H20C	−0.155946	0.067977	0.393803	0.137*	
C21A	0.1855 (5)	0.5910 (3)	0.3088 (2)	0.0614 (10)	
C22A	0.0849 (4)	0.5601 (3)	0.3784 (2)	0.0632 (11)	
H22A	0.090530	0.485735	0.394411	0.076*	
H22B	−0.012388	0.578229	0.362596	0.076*	
C23A	0.0510 (5)	0.7366 (4)	0.4305 (3)	0.0786 (13)	
S1B	0.31199 (16)	0.38092 (12)	0.54897 (10)	0.1127 (6)	
O1B'	0.566 (3)	0.190 (4)	0.947 (2)	0.113 (12)	0.42 (12)
O1B"	0.539 (4)	0.131 (8)	0.970 (3)	0.171 (18)	0.58 (12)
O2B	0.1951 (4)	0.3255 (3)	0.69262 (19)	0.0931 (10)	
N1B	0.3482 (3)	0.2068 (3)	0.7563 (2)	0.0683 (9)	
N2B	0.5088 (7)	0.3965 (5)	0.4222 (3)	0.138 (2)	
C1B	0.2562 (5)	0.1995 (4)	0.8278 (3)	0.0818 (13)	
H1B	0.184797	0.255715	0.817285	0.098*	
C2B	0.3475 (6)	0.2238 (5)	0.8937 (3)	0.113 (2)	
H2B1	0.372967	0.295922	0.882092	0.135*	
H2B2	0.290958	0.212634	0.942081	0.135*	
C3B	0.4784 (7)	0.1601 (6)	0.9040 (3)	0.117 (2)	
C4B	0.5166 (5)	0.0956 (4)	0.8439 (3)	0.0867 (14)	
H4B	0.452076	0.036950	0.853003	0.104*	
C5B	0.4925 (4)	0.1539 (3)	0.7612 (2)	0.0683 (11)	
H5B	0.564398	0.207852	0.749835	0.082*	
C6B	0.6656 (7)	0.0527 (7)	0.8517 (4)	0.161 (3)	
H6B1	0.685894	0.011937	0.812203	0.241*	
H6B2	0.731887	0.108273	0.845718	0.241*	
H6B3	0.675086	0.010353	0.902242	0.241*	
C7B	0.1730 (5)	0.1004 (4)	0.8438 (3)	0.0743 (12)	
C8B	0.1751 (6)	0.0319 (5)	0.9134 (3)	0.1013 (17)	
H8B	0.232060	0.044863	0.953041	0.122*	
C9B	0.0933 (7)	−0.0562 (5)	0.9251 (3)	0.1070 (19)	
H9B	0.096268	−0.100407	0.973113	0.128*	
C10B	0.0078 (6)	−0.0812 (4)	0.8688 (3)	0.0907 (15)	
C11B	0.0082 (5)	−0.0112 (4)	0.7989 (3)	0.0859 (14)	
H11B	−0.046863	−0.024349	0.758613	0.103*	
C12B	0.0874 (5)	0.0768 (4)	0.7876 (3)	0.0806 (13)	
H12B	0.082761	0.122016	0.740095	0.097*	
C13B	−0.0806 (8)	−0.1760 (5)	0.8814 (4)	0.131 (2)	
H13D	−0.131733	−0.178588	0.835043	0.197*	
H13E	−0.019257	−0.235928	0.893103	0.197*	
H13F	−0.147728	−0.174285	0.924106	0.197*	
C14B	0.5181 (4)	0.0832 (3)	0.7030 (2)	0.0600 (10)	
C15B	0.6424 (5)	0.0907 (3)	0.6581 (3)	0.0725 (12)	
H15B	0.705753	0.143012	0.661186	0.087*	
C16B	0.6754 (5)	0.0229 (4)	0.6087 (3)	0.0798 (13)	
H16B	0.760980	0.029772	0.579453	0.096*	
C17B	0.5853 (6)	−0.0543 (4)	0.6016 (3)	0.0843 (14)	
C18B	0.4602 (6)	−0.0629 (4)	0.6471 (3)	0.0899 (15)	
H18B	0.397403	−0.115546	0.644031	0.108*	
C19B	0.4262 (5)	0.0057 (3)	0.6975 (3)	0.0755 (12)	
H19B	0.341369	−0.001190	0.727332	0.091*	
C20B	0.6186 (7)	−0.1281 (5)	0.5459 (3)	0.128 (2)	
H20D	0.542830	−0.176645	0.549288	0.192*	
H20E	0.627003	−0.089979	0.493608	0.192*	
H20F	0.707204	−0.164370	0.559473	0.192*	
C21B	0.3082 (5)	0.2761 (4)	0.6935 (3)	0.0705 (12)	
C22B	0.4038 (5)	0.2918 (4)	0.6211 (3)	0.0782 (13)	
H22C	0.421408	0.226851	0.603442	0.094*	
H22D	0.494952	0.319238	0.631557	0.094*	
C23B	0.4310 (7)	0.3895 (4)	0.4752 (4)	0.0989 (16)	

### 1-[2-(Cyanosulfanyl)acetyl]-3-methyl-2,6-bis(4-methylphenyl)piperidin-4-one Atomic displacement parameters (Å^2^)

**Table:**

	*U*^11^	*U*^22^	*U*^33^	*U*^12^	*U*^13^	*U*^23^
S1A	0.1036 (9)	0.0730 (8)	0.0638 (7)	0.0026 (6)	−0.0158 (6)	−0.0203 (6)
O1A	0.148 (4)	0.203 (5)	0.109 (3)	0.008 (3)	−0.046 (3)	0.066 (3)
O2A	0.085 (2)	0.088 (2)	0.092 (2)	−0.0289 (19)	0.0028 (17)	−0.0259 (18)
N1A	0.069 (2)	0.0524 (19)	0.0539 (19)	−0.0043 (16)	0.0046 (16)	−0.0064 (15)
N2A	0.190 (5)	0.070 (3)	0.106 (4)	0.007 (3)	−0.019 (3)	−0.025 (3)
C1A	0.082 (3)	0.072 (3)	0.064 (3)	−0.006 (2)	0.004 (2)	0.000 (2)
C2A	0.116 (4)	0.084 (3)	0.067 (3)	0.011 (3)	0.011 (3)	0.017 (3)
C3A	0.096 (4)	0.106 (4)	0.067 (3)	0.019 (3)	−0.006 (3)	0.007 (3)
C4A	0.072 (3)	0.079 (3)	0.058 (3)	0.010 (2)	−0.011 (2)	−0.011 (2)
C5A	0.061 (2)	0.053 (2)	0.053 (2)	0.0078 (19)	−0.0071 (18)	−0.0064 (18)
C6A	0.129 (5)	0.160 (6)	0.092 (4)	−0.007 (4)	−0.022 (4)	−0.023 (4)
C7A	0.069 (3)	0.086 (3)	0.057 (3)	−0.005 (2)	0.010 (2)	−0.004 (2)
C8A	0.098 (4)	0.138 (5)	0.053 (3)	0.019 (4)	0.003 (2)	−0.017 (3)
C9A	0.110 (4)	0.142 (5)	0.069 (3)	0.019 (4)	0.004 (3)	−0.040 (3)
C10A	0.083 (3)	0.108 (4)	0.076 (3)	0.013 (3)	0.006 (3)	−0.023 (3)
C11A	0.070 (3)	0.112 (4)	0.071 (3)	0.007 (3)	−0.009 (2)	−0.017 (3)
C12A	0.069 (3)	0.099 (4)	0.061 (3)	0.002 (3)	−0.001 (2)	−0.020 (3)
C13A	0.136 (5)	0.138 (5)	0.134 (5)	0.040 (4)	−0.005 (4)	−0.052 (4)
C14A	0.056 (2)	0.048 (2)	0.053 (2)	0.0017 (19)	−0.0058 (18)	−0.0139 (18)
C15A	0.058 (3)	0.058 (3)	0.063 (2)	0.006 (2)	−0.006 (2)	−0.016 (2)
C16A	0.068 (3)	0.062 (3)	0.064 (3)	−0.010 (2)	0.003 (2)	−0.017 (2)
C17A	0.075 (3)	0.057 (3)	0.065 (3)	−0.005 (2)	−0.010 (2)	−0.008 (2)
C18A	0.068 (3)	0.056 (3)	0.085 (3)	0.013 (2)	−0.012 (2)	−0.003 (2)
C19A	0.059 (3)	0.057 (3)	0.076 (3)	0.006 (2)	−0.002 (2)	−0.008 (2)
C20A	0.110 (4)	0.064 (3)	0.094 (3)	−0.008 (3)	−0.004 (3)	0.005 (3)
C21A	0.071 (3)	0.047 (2)	0.066 (3)	0.000 (2)	−0.002 (2)	−0.009 (2)
C22A	0.076 (3)	0.052 (2)	0.064 (2)	0.005 (2)	−0.008 (2)	−0.0151 (19)
C23A	0.109 (4)	0.059 (3)	0.073 (3)	−0.011 (3)	−0.003 (3)	−0.023 (2)
S1B	0.1023 (11)	0.0932 (10)	0.1248 (12)	0.0301 (8)	0.0101 (9)	0.0202 (9)
O1B'	0.107 (11)	0.17 (3)	0.072 (12)	−0.009 (10)	−0.014 (8)	−0.048 (12)
O1B"	0.138 (11)	0.30 (5)	0.095 (13)	0.01 (2)	−0.033 (9)	−0.08 (2)
O2B	0.081 (2)	0.088 (2)	0.112 (3)	0.0324 (19)	0.0037 (18)	−0.0274 (19)
N1B	0.058 (2)	0.082 (2)	0.069 (2)	0.0078 (18)	0.0014 (18)	−0.028 (2)
N2B	0.140 (5)	0.161 (5)	0.103 (4)	−0.019 (4)	0.004 (4)	0.002 (4)
C1B	0.070 (3)	0.106 (4)	0.074 (3)	0.015 (3)	0.005 (2)	−0.033 (3)
C2B	0.097 (4)	0.173 (6)	0.084 (4)	0.008 (4)	0.003 (3)	−0.068 (4)
C3B	0.099 (4)	0.187 (6)	0.078 (4)	0.007 (4)	−0.016 (3)	−0.059 (4)
C4B	0.068 (3)	0.120 (4)	0.073 (3)	0.019 (3)	−0.016 (2)	−0.021 (3)
C5B	0.054 (2)	0.079 (3)	0.076 (3)	0.004 (2)	−0.003 (2)	−0.026 (2)
C6B	0.123 (5)	0.238 (9)	0.122 (5)	0.059 (6)	−0.033 (4)	−0.038 (6)
C7B	0.065 (3)	0.105 (4)	0.053 (3)	0.012 (3)	0.005 (2)	−0.017 (3)
C8B	0.099 (4)	0.138 (5)	0.068 (4)	0.003 (4)	−0.003 (3)	−0.022 (4)
C9B	0.125 (5)	0.120 (5)	0.065 (3)	0.028 (4)	0.005 (3)	0.005 (3)
C10B	0.101 (4)	0.092 (4)	0.074 (3)	0.010 (3)	0.018 (3)	−0.006 (3)
C11B	0.086 (3)	0.102 (4)	0.068 (3)	−0.003 (3)	−0.001 (2)	−0.009 (3)
C12B	0.080 (3)	0.096 (4)	0.060 (3)	−0.002 (3)	0.000 (2)	0.000 (3)
C13B	0.179 (6)	0.099 (4)	0.110 (5)	−0.015 (4)	0.022 (4)	−0.002 (4)
C14B	0.055 (2)	0.059 (2)	0.067 (3)	0.006 (2)	−0.008 (2)	−0.014 (2)
C15B	0.069 (3)	0.070 (3)	0.081 (3)	0.006 (2)	−0.001 (2)	−0.020 (2)
C16B	0.079 (3)	0.079 (3)	0.080 (3)	0.018 (3)	0.007 (2)	−0.016 (3)
C17B	0.092 (4)	0.079 (3)	0.087 (3)	0.036 (3)	−0.025 (3)	−0.027 (3)
C18B	0.087 (4)	0.068 (3)	0.122 (4)	0.007 (3)	−0.032 (3)	−0.034 (3)
C19B	0.064 (3)	0.074 (3)	0.091 (3)	0.001 (2)	−0.004 (2)	−0.021 (3)
C20B	0.157 (5)	0.114 (4)	0.131 (5)	0.058 (4)	−0.052 (4)	−0.072 (4)
C21B	0.069 (3)	0.069 (3)	0.078 (3)	0.007 (2)	−0.003 (2)	−0.027 (3)
C22B	0.072 (3)	0.080 (3)	0.084 (3)	0.005 (2)	−0.009 (2)	−0.017 (3)
C23B	0.104 (4)	0.083 (4)	0.101 (4)	−0.006 (3)	−0.003 (3)	0.008 (3)

### 1-[2-(Cyanosulfanyl)acetyl]-3-methyl-2,6-bis(4-methylphenyl)piperidin-4-one Geometric parameters (Å, º)

**Table:**

S1A—C23A	1.670 (6)	S1B—C22B	1.785 (4)
S1A—C22A	1.802 (4)	O1B'—C3B	1.25 (2)
O1A—C3A	1.206 (6)	O1B"—C3B	1.29 (4)
O2A—C21A	1.221 (5)	O2B—C21B	1.226 (5)
N1A—C21A	1.357 (5)	N1B—C21B	1.350 (5)
N1A—C5A	1.484 (5)	N1B—C1B	1.481 (5)
N1A—C1A	1.490 (5)	N1B—C5B	1.499 (5)
N2A—C23A	1.133 (6)	N2B—C23B	1.149 (7)
C1A—C7A	1.525 (6)	C1B—C7B	1.515 (7)
C1A—C2A	1.538 (6)	C1B—C2B	1.539 (7)
C1A—H1A	0.9800	C1B—H1B	0.9800
C2A—C3A	1.475 (7)	C2B—C3B	1.463 (8)
C2A—H2A1	0.9700	C2B—H2B1	0.9700
C2A—H2A2	0.9700	C2B—H2B2	0.9700
C3A—C4A	1.493 (7)	C3B—C4B	1.494 (7)
C4A—C6A	1.475 (7)	C4B—C6B	1.490 (7)
C4A—C5A	1.548 (5)	C4B—C5B	1.529 (6)
C4A—H4A	0.9800	C4B—H4B	0.9800
C5A—C14A	1.510 (5)	C5B—C14B	1.509 (5)
C5A—H5A	0.9800	C5B—H5B	0.9800
C6A—H6A1	0.9600	C6B—H6B1	0.9600
C6A—H6A2	0.9600	C6B—H6B2	0.9600
C6A—H6A3	0.9600	C6B—H6B3	0.9600
C7A—C8A	1.369 (6)	C7B—C12B	1.371 (6)
C7A—C12A	1.389 (6)	C7B—C8B	1.376 (7)
C8A—C9A	1.379 (7)	C8B—C9B	1.388 (8)
C8A—H8A	0.9300	C8B—H8B	0.9300
C9A—C10A	1.373 (7)	C9B—C10B	1.378 (8)
C9A—H9A	0.9300	C9B—H9B	0.9300
C10A—C11A	1.382 (6)	C10B—C11B	1.389 (7)
C10A—C13A	1.508 (7)	C10B—C13B	1.494 (8)
C11A—C12A	1.385 (6)	C11B—C12B	1.373 (7)
C11A—H11A	0.9300	C11B—H11B	0.9300
C12A—H12A	0.9300	C12B—H12B	0.9300
C13A—H13A	0.9600	C13B—H13D	0.9600
C13A—H13B	0.9600	C13B—H13E	0.9600
C13A—H13C	0.9600	C13B—H13F	0.9600
C14A—C19A	1.385 (5)	C14B—C19B	1.369 (6)
C14A—C15A	1.386 (5)	C14B—C15B	1.374 (5)
C15A—C16A	1.377 (5)	C15B—C16B	1.374 (6)
C15A—H15A	0.9300	C15B—H15B	0.9300
C16A—C17A	1.374 (6)	C16B—C17B	1.364 (7)
C16A—H16A	0.9300	C16B—H16B	0.9300
C17A—C18A	1.382 (6)	C17B—C18B	1.385 (7)
C17A—C20A	1.512 (6)	C17B—C20B	1.515 (7)
C18A—C19A	1.383 (6)	C18B—C19B	1.396 (6)
C18A—H18A	0.9300	C18B—H18B	0.9300
C19A—H19A	0.9300	C19B—H19B	0.9300
C20A—H20A	0.9600	C20B—H20D	0.9600
C20A—H20B	0.9600	C20B—H20E	0.9600
C20A—H20C	0.9600	C20B—H20F	0.9600
C21A—C22A	1.512 (5)	C21B—C22B	1.507 (6)
C22A—H22A	0.9700	C22B—H22C	0.9700
C22A—H22B	0.9700	C22B—H22D	0.9700
S1B—C23B	1.660 (7)		
			
C23A—S1A—C22A	99.8 (2)	C21B—N1B—C1B	117.1 (3)
C21A—N1A—C5A	122.9 (3)	C21B—N1B—C5B	122.8 (4)
C21A—N1A—C1A	116.7 (3)	C1B—N1B—C5B	119.0 (3)
C5A—N1A—C1A	119.9 (3)	N1B—C1B—C7B	111.6 (4)
N1A—C1A—C7A	111.4 (3)	N1B—C1B—C2B	108.4 (4)
N1A—C1A—C2A	107.9 (3)	C7B—C1B—C2B	117.5 (4)
C7A—C1A—C2A	116.9 (4)	N1B—C1B—H1B	106.2
N1A—C1A—H1A	106.7	C7B—C1B—H1B	106.2
C7A—C1A—H1A	106.7	C2B—C1B—H1B	106.2
C2A—C1A—H1A	106.7	C3B—C2B—C1B	112.5 (4)
C3A—C2A—C1A	115.0 (4)	C3B—C2B—H2B1	109.1
C3A—C2A—H2A1	108.5	C1B—C2B—H2B1	109.1
C1A—C2A—H2A1	108.5	C3B—C2B—H2B2	109.1
C3A—C2A—H2A2	108.5	C1B—C2B—H2B2	109.1
C1A—C2A—H2A2	108.5	H2B1—C2B—H2B2	107.8
H2A1—C2A—H2A2	107.5	O1B'—C3B—C2B	113.5 (15)
O1A—C3A—C2A	121.7 (5)	O1B"—C3B—C2B	124.3 (12)
O1A—C3A—C4A	122.1 (6)	O1B'—C3B—C4B	124.8 (12)
C2A—C3A—C4A	116.2 (4)	O1B"—C3B—C4B	115 (3)
C6A—C4A—C3A	110.3 (4)	C2B—C3B—C4B	117.7 (5)
C6A—C4A—C5A	112.1 (4)	C6B—C4B—C3B	112.1 (5)
C3A—C4A—C5A	111.6 (4)	C6B—C4B—C5B	111.1 (4)
C6A—C4A—H4A	107.6	C3B—C4B—C5B	112.1 (4)
C3A—C4A—H4A	107.6	C6B—C4B—H4B	107.1
C5A—C4A—H4A	107.6	C3B—C4B—H4B	107.1
N1A—C5A—C14A	114.3 (3)	C5B—C4B—H4B	107.1
N1A—C5A—C4A	110.9 (3)	N1B—C5B—C14B	113.9 (3)
C14A—C5A—C4A	108.7 (3)	N1B—C5B—C4B	110.6 (3)
N1A—C5A—H5A	107.5	C14B—C5B—C4B	110.4 (4)
C14A—C5A—H5A	107.5	N1B—C5B—H5B	107.2
C4A—C5A—H5A	107.5	C14B—C5B—H5B	107.2
C4A—C6A—H6A1	109.5	C4B—C5B—H5B	107.2
C4A—C6A—H6A2	109.5	C4B—C6B—H6B1	109.5
H6A1—C6A—H6A2	109.5	C4B—C6B—H6B2	109.5
C4A—C6A—H6A3	109.5	H6B1—C6B—H6B2	109.5
H6A1—C6A—H6A3	109.5	C4B—C6B—H6B3	109.5
H6A2—C6A—H6A3	109.5	H6B1—C6B—H6B3	109.5
C8A—C7A—C12A	117.2 (4)	H6B2—C6B—H6B3	109.5
C8A—C7A—C1A	125.7 (4)	C12B—C7B—C8B	116.8 (5)
C12A—C7A—C1A	117.0 (4)	C12B—C7B—C1B	119.6 (4)
C7A—C8A—C9A	121.5 (5)	C8B—C7B—C1B	123.6 (5)
C7A—C8A—H8A	119.2	C7B—C8B—C9B	120.8 (5)
C9A—C8A—H8A	119.2	C7B—C8B—H8B	119.6
C10A—C9A—C8A	122.1 (5)	C9B—C8B—H8B	119.6
C10A—C9A—H9A	119.0	C10B—C9B—C8B	122.9 (5)
C8A—C9A—H9A	119.0	C10B—C9B—H9B	118.6
C9A—C10A—C11A	116.6 (5)	C8B—C9B—H9B	118.6
C9A—C10A—C13A	122.0 (5)	C9B—C10B—C11B	115.2 (5)
C11A—C10A—C13A	121.4 (5)	C9B—C10B—C13B	123.0 (5)
C10A—C11A—C12A	121.8 (4)	C11B—C10B—C13B	121.9 (6)
C10A—C11A—H11A	119.1	C12B—C11B—C10B	122.1 (5)
C12A—C11A—H11A	119.1	C12B—C11B—H11B	119.0
C11A—C12A—C7A	120.8 (4)	C10B—C11B—H11B	119.0
C11A—C12A—H12A	119.6	C7B—C12B—C11B	122.2 (5)
C7A—C12A—H12A	119.6	C7B—C12B—H12B	118.9
C10A—C13A—H13A	109.5	C11B—C12B—H12B	118.9
C10A—C13A—H13B	109.5	C10B—C13B—H13D	109.5
H13A—C13A—H13B	109.5	C10B—C13B—H13E	109.5
C10A—C13A—H13C	109.5	H13D—C13B—H13E	109.5
H13A—C13A—H13C	109.5	C10B—C13B—H13F	109.5
H13B—C13A—H13C	109.5	H13D—C13B—H13F	109.5
C19A—C14A—C15A	117.6 (3)	H13E—C13B—H13F	109.5
C19A—C14A—C5A	121.9 (3)	C19B—C14B—C15B	118.3 (4)
C15A—C14A—C5A	120.3 (3)	C19B—C14B—C5B	122.0 (4)
C16A—C15A—C14A	121.2 (4)	C15B—C14B—C5B	119.5 (4)
C16A—C15A—H15A	119.4	C16B—C15B—C14B	121.5 (4)
C14A—C15A—H15A	119.4	C16B—C15B—H15B	119.2
C17A—C16A—C15A	121.3 (4)	C14B—C15B—H15B	119.2
C17A—C16A—H16A	119.4	C17B—C16B—C15B	121.3 (4)
C15A—C16A—H16A	119.4	C17B—C16B—H16B	119.3
C16A—C17A—C18A	117.8 (4)	C15B—C16B—H16B	119.3
C16A—C17A—C20A	121.4 (4)	C16B—C17B—C18B	117.5 (4)
C18A—C17A—C20A	120.8 (4)	C16B—C17B—C20B	121.9 (5)
C17A—C18A—C19A	121.2 (4)	C18B—C17B—C20B	120.6 (5)
C17A—C18A—H18A	119.4	C17B—C18B—C19B	121.4 (5)
C19A—C18A—H18A	119.4	C17B—C18B—H18B	119.3
C18A—C19A—C14A	120.8 (4)	C19B—C18B—H18B	119.3
C18A—C19A—H19A	119.6	C14B—C19B—C18B	119.9 (4)
C14A—C19A—H19A	119.6	C14B—C19B—H19B	120.0
C17A—C20A—H20A	109.5	C18B—C19B—H19B	120.0
C17A—C20A—H20B	109.5	C17B—C20B—H20D	109.5
H20A—C20A—H20B	109.5	C17B—C20B—H20E	109.5
C17A—C20A—H20C	109.5	H20D—C20B—H20E	109.5
H20A—C20A—H20C	109.5	C17B—C20B—H20F	109.5
H20B—C20A—H20C	109.5	H20D—C20B—H20F	109.5
O2A—C21A—N1A	122.8 (4)	H20E—C20B—H20F	109.5
O2A—C21A—C22A	120.2 (4)	O2B—C21B—N1B	122.3 (4)
N1A—C21A—C22A	116.9 (4)	O2B—C21B—C22B	118.5 (4)
C21A—C22A—S1A	114.3 (3)	N1B—C21B—C22B	119.1 (4)
C21A—C22A—H22A	108.7	C21B—C22B—S1B	106.7 (3)
S1A—C22A—H22A	108.7	C21B—C22B—H22C	110.4
C21A—C22A—H22B	108.7	S1B—C22B—H22C	110.4
S1A—C22A—H22B	108.7	C21B—C22B—H22D	110.4
H22A—C22A—H22B	107.6	S1B—C22B—H22D	110.4
N2A—C23A—S1A	177.9 (5)	H22C—C22B—H22D	108.6
C23B—S1B—C22B	100.1 (2)	N2B—C23B—S1B	177.2 (6)
			
C21A—N1A—C1A—C7A	106.1 (4)	C21B—N1B—C1B—C2B	−120.5 (5)
C5A—N1A—C1A—C7A	−81.7 (4)	C5B—N1B—C1B—C2B	47.7 (5)
C21A—N1A—C1A—C2A	−124.2 (4)	N1B—C1B—C2B—C3B	−53.2 (7)
C5A—N1A—C1A—C2A	47.9 (5)	C7B—C1B—C2B—C3B	74.5 (6)
N1A—C1A—C2A—C3A	−47.9 (5)	C1B—C2B—C3B—O1B'	168 (3)
C7A—C1A—C2A—C3A	78.6 (5)	C1B—C2B—C3B—O1B"	−149 (6)
C1A—C2A—C3A—O1A	−178.3 (5)	C1B—C2B—C3B—C4B	9.5 (8)
C1A—C2A—C3A—C4A	2.1 (6)	O1B'—C3B—C4B—C6B	12 (4)
O1A—C3A—C4A—C6A	−7.6 (7)	O1B"—C3B—C4B—C6B	−32 (5)
C2A—C3A—C4A—C6A	172.0 (5)	C2B—C3B—C4B—C6B	167.8 (6)
O1A—C3A—C4A—C5A	−132.8 (6)	O1B'—C3B—C4B—C5B	−114 (4)
C2A—C3A—C4A—C5A	46.7 (6)	O1B"—C3B—C4B—C5B	−157 (5)
C21A—N1A—C5A—C14A	−66.4 (4)	C2B—C3B—C4B—C5B	42.1 (7)
C1A—N1A—C5A—C14A	122.0 (3)	C21B—N1B—C5B—C14B	−65.4 (5)
C21A—N1A—C5A—C4A	170.3 (3)	C1B—N1B—C5B—C14B	127.2 (4)
C1A—N1A—C5A—C4A	−1.4 (4)	C21B—N1B—C5B—C4B	169.6 (4)
C6A—C4A—C5A—N1A	−171.0 (4)	C1B—N1B—C5B—C4B	2.1 (5)
C3A—C4A—C5A—N1A	−46.7 (5)	C6B—C4B—C5B—N1B	−173.6 (5)
C6A—C4A—C5A—C14A	62.5 (5)	C3B—C4B—C5B—N1B	−47.4 (5)
C3A—C4A—C5A—C14A	−173.2 (4)	C6B—C4B—C5B—C14B	59.4 (6)
N1A—C1A—C7A—C8A	122.0 (5)	C3B—C4B—C5B—C14B	−174.4 (4)
C2A—C1A—C7A—C8A	−2.8 (7)	N1B—C1B—C7B—C12B	−55.5 (5)
N1A—C1A—C7A—C12A	−59.1 (5)	C2B—C1B—C7B—C12B	178.4 (4)
C2A—C1A—C7A—C12A	176.2 (4)	N1B—C1B—C7B—C8B	126.0 (5)
C12A—C7A—C8A—C9A	2.2 (7)	C2B—C1B—C7B—C8B	0.0 (6)
C1A—C7A—C8A—C9A	−178.9 (5)	C12B—C7B—C8B—C9B	−0.3 (7)
C7A—C8A—C9A—C10A	−0.4 (9)	C1B—C7B—C8B—C9B	178.2 (5)
C8A—C9A—C10A—C11A	−1.3 (8)	C7B—C8B—C9B—C10B	0.9 (8)
C8A—C9A—C10A—C13A	178.7 (6)	C8B—C9B—C10B—C11B	−0.4 (8)
C9A—C10A—C11A—C12A	1.2 (7)	C8B—C9B—C10B—C13B	−179.8 (5)
C13A—C10A—C11A—C12A	−178.8 (5)	C9B—C10B—C11B—C12B	−0.6 (7)
C10A—C11A—C12A—C7A	0.6 (7)	C13B—C10B—C11B—C12B	178.9 (5)
C8A—C7A—C12A—C11A	−2.2 (7)	C8B—C7B—C12B—C11B	−0.7 (7)
C1A—C7A—C12A—C11A	178.7 (4)	C1B—C7B—C12B—C11B	−179.2 (4)
N1A—C5A—C14A—C19A	−52.6 (5)	C10B—C11B—C12B—C7B	1.2 (7)
C4A—C5A—C14A—C19A	71.9 (4)	N1B—C5B—C14B—C19B	−54.5 (5)
N1A—C5A—C14A—C15A	132.6 (3)	C4B—C5B—C14B—C19B	70.6 (5)
C4A—C5A—C14A—C15A	−102.8 (4)	N1B—C5B—C14B—C15B	130.7 (4)
C19A—C14A—C15A—C16A	−1.2 (5)	C4B—C5B—C14B—C15B	−104.2 (4)
C5A—C14A—C15A—C16A	173.8 (3)	C19B—C14B—C15B—C16B	0.0 (6)
C14A—C15A—C16A—C17A	−0.3 (6)	C5B—C14B—C15B—C16B	174.9 (4)
C15A—C16A—C17A—C18A	1.7 (6)	C14B—C15B—C16B—C17B	0.6 (7)
C15A—C16A—C17A—C20A	−179.6 (4)	C15B—C16B—C17B—C18B	−1.1 (7)
C16A—C17A—C18A—C19A	−1.6 (6)	C15B—C16B—C17B—C20B	178.5 (4)
C20A—C17A—C18A—C19A	179.7 (4)	C16B—C17B—C18B—C19B	0.9 (7)
C17A—C18A—C19A—C14A	0.1 (6)	C20B—C17B—C18B—C19B	−178.6 (4)
C15A—C14A—C19A—C18A	1.3 (6)	C15B—C14B—C19B—C18B	−0.1 (6)
C5A—C14A—C19A—C18A	−173.6 (4)	C5B—C14B—C19B—C18B	−174.9 (4)
C5A—N1A—C21A—O2A	−174.1 (3)	C17B—C18B—C19B—C14B	−0.4 (7)
C1A—N1A—C21A—O2A	−2.2 (5)	C1B—N1B—C21B—O2B	−5.8 (6)
C5A—N1A—C21A—C22A	8.5 (5)	C5B—N1B—C21B—O2B	−173.5 (4)
C1A—N1A—C21A—C22A	−179.6 (3)	C1B—N1B—C21B—C22B	175.7 (4)
O2A—C21A—C22A—S1A	9.6 (5)	C5B—N1B—C21B—C22B	8.0 (6)
N1A—C21A—C22A—S1A	−172.9 (3)	O2B—C21B—C22B—S1B	−2.5 (5)
C23A—S1A—C22A—C21A	77.3 (3)	N1B—C21B—C22B—S1B	176.0 (3)
C21B—N1B—C1B—C7B	108.6 (4)	C23B—S1B—C22B—C21B	179.5 (3)
C5B—N1B—C1B—C7B	−83.3 (5)		

### 1-[2-(Cyanosulfanyl)acetyl]-3-methyl-2,6-bis(4-methylphenyl)piperidin-4-one Hydrogen-bond geometry (Å, º)

**Table:**

*D*—H···*A*	*D*—H	H···*A*	*D*···*A*	*D*—H···*A*
C1*A*—H1*A*···O2*A*	0.98	2.22	2.706 (5)	109
C1*B*—H1*B*···O2*B*	0.98	2.20	2.697 (6)	110
C5*A*—H5*A*···O2*B*^i^	0.98	2.40	3.345 (5)	161
C5*B*—H5*B*···O2*A*^ii^	0.98	2.51	3.458 (5)	163
C22*A*—H22*B*···O2*B*^i^	0.97	2.24	3.162 (5)	159
C22*B*—H22*D*···O2*A*^ii^	0.97	2.34	3.293 (5)	166

Symmetry codes: (i) −*x*, −*y*+1, −*z*+1; (ii) −*x*+1, −*y*+1, −*z*+1.

### Global reactivity parameters of (I)

**Table:**

Parameters (eV)	B3LYP/6311G++(d,p)
Energy (a.u)	-1549.3657
Energy	-42160.4098
HOMO	-6.805
LUMO	-1.6463
Energy gap	5.1587
Ionization potential (IP)	6.805
electron affinity (EA)	1.6463
Absolute electronegativity (χ)	4.2257
global softness (σ)	0.3877
global hardness (η)	2.5794
electronic chemical potential (µ)	-4.2257
electrophilicity index (ω)	6.9227
